# Super resolution measurement of collagen fibers in biological samples: Validation of a commercial solution for multiphoton microscopy

**DOI:** 10.1371/journal.pone.0229278

**Published:** 2020-02-14

**Authors:** Aaron M. Barlow, Leila B. Mostaço-Guidolin, Emmanuel T. Osei, Steven Booth, Tillie-Louise Hackett

**Affiliations:** 1 Centre for Heart Lung Innovation, St. Paul’s Hospital, Vancouver, BC, Canada; 2 Department of Anesthesiology, Pharmacology and Therapeutics, University of British Columbia, Vancouver, BC, Canada; 3 Department of Systems and Computer Engineering, Carleton University, Ottawa, ON, Canada; Illinois Institute of Technology, UNITED STATES

## Abstract

Multiphoton microscopy is a powerful, non-invasive technique to image biological specimens. One current limitation of multiphoton microscopy is resolution as many of the biological molecules and structures investigated by research groups are similar in size or smaller than the diffraction limit. To date, the combination of multiphoton and super-resolution imaging has proved technically challenging for biology focused laboratories to implement. Here we validate that the commercial super-resolution Airyscan detector from ZEISS, which is based on image scanning microscopy, can be integrated under warranty with a pulsed multi-photon laser to enable multiphoton microscopy with super-resolution. We demonstrate its biological application in two different imaging modalities, second harmonic generation (SHG) and two-photon excited fluorescence (TPEF), to measure the fibre thicknesses of collagen and elastin molecules surpassing the diffraction limit by a factor of 1.7±0.3x and 1.4±0.3x respectively, in human heart and lung tissues, and 3-dimensional *in vitro* models. We show that enhanced resolution and signal-to-noise of SHG using the Airyscan compared to traditional GaAs detectors allows for automated and precise measurement of collagen fibres using texture analysis in biological tissues.

## Introduction

Multiphoton microscopy has become the method of choice for imaging live, intact biological tissues due to the advantages of depth penetration and reduced photodamage, as a result of employing a near infrared femtosecond laser to generate observable nonlinear signals in the visible range [1–4]. Multiphoton excitation occurs when two (or more) photons arrive simultaneously at a fluorophore, and their energy sum satisfies the transition energy required to promote the fluorophore from a ground to an excited state. Such two-photon excitation fluorescence (TPEF) can be generated from a range of applications including exogenous probes (e.g. Hoechst), transfected proteins (e.g. Green fluorescent protein) or endogenous tissue molecules (e.g. NAD(P)H). Multiphoton imaging is also sensitive to second harmonic generation (SHG) where two photons instantaneously convert their energy into a single photon of half the wavelength of the original pair, which can occur in highly ordered, non-centrosymmetric structures [5], including biomolecules such as fibrillar collagen. Fibrillar collagens are the predominant protein in the human body, being the principal component of connective tissues. Fibrillar collagen is important for providing the tensile strength of tissues, while other extracellular matrix (ECM) molecules, such as elastin provide elasticity to tissues during repetitive strain and relaxation, such as in the breathing lung or the beating heart [6]. Alterations in the concentration and distribution of collagen and elastin fibers can lead to loss of anatomical structure, compromised function, and tissue fibrosis [7,8]. It is difficult to study the alterations in the packaging and arrangement of ECM fibers that can occur with disease using conventional light microscopy or multiphoton microscopy, since these techniques are limited by the diffraction limit (~200–300 nm in the lateral direction and 500–700 nm in the transverse direction), which is similar in size or larger than many biological structures, including fibrillar collagen and elastin [9,10].

To exceed the conventional diffraction limit, researchers have developed a suite of optical imaging techniques collectively known as super-resolution (SR) imaging [11–14]. While these techniques have been effective for enhancing the resolution of confocal microscopes, many of them do so by using specialized flurophores or excitation techniques that do not have obvious multiphoton counterparts [7,15,16]. One SR-multiphoton alternative that avoids these problems is imaging scanning microscopy (ISM). ISM is a detection-based technique where the point-spread function (PSF) of the diffracted light from sub-resolution objects are collected, and this additional information is used to improve the resolution of the resulting image [16–21]. The effect of combining ISM with two-photon fluorescence microscopy has been evaluated in detail by Sheppard et al [22], and SR-MP has been implemented by Gregor et al [17], who demonstrated an all-optical ISM design that allows straightforward implementation into existing microscopes. However, all SR-MP systems currently require custom-built or heavily modified systems, which are difficult to implement for biology focused laboratories.

In this report, we demonstrate the feasibility of using the commercially available Airyscan system to perform endogenous simultaneous super resolution-SHG and TPEF image acquisition using a pulsed laser source, and its utility to study the structures of fibrillar collagen and elastin fibers in human tissues and 3-dimensional *in vitro* models. The Airyscan detection system developed by Zeiss is an ISM module that uses a unique detector design coupled to a confocal microscope to achieve super resolution [23]. Traditional confocal laser-scanning microscopes use a physical aperture for a pinhole and single point-detector. In comparison, the Airyscan uses a 32-channel gallium arsenide phosphide photomultiplier tube (GaAsP-PMT) area detector, which enables a large pinhole collection efficiency of 1.25 Airy units (AU), but with an improved signal-to-noise ratio (SNR) as each detector channel functions as a single small pinhole of 0.2AU. The resulting resolution can exceed the confocal diffraction limit by a factor of 1.7x. SR microscopy with the Airyscan has been used primarily to enhance the resolution of traditional confocal microscopy [24], live cell imaging [25], and FRET [26]. A recent commercial report published by Zeiss has also demonstrated the feasibility of using a pulsed laser source to generate two-photon excitation in fluorescently labelled tissues [27].

We report here that the Airyscan when compared to standard MP microscopy for SHG and TPEF **s**urpasses the diffraction limit by a factor of 1.7±0.3x and 1.4±0.3x, respectively, when measuring the fibre thicknesses of collagen and elastin molecules in human tissues and 3-dimentional *in vitro* models. We also demonstrate that the improved resolution and SNR afforded by the Airsycan improves the quality and accuracy of texture analysis to assess the structural integrity of fibrillar collagen.

### Optical properties

The optical properties of multiphoton microscopy are quite different from confocal microscopy, as the multiphoton response scales nonlinearly with the intensity of the laser source. Detectable signal is only achieved within the Rayleigh range of the laser focus, which acts as an effective pinhole whose resolution depends on the intensity of the incident light. However, the focal volume generated by the laser focus is an ellipsoid with its long axis oriented in the direction of laser propagation. As a result, in an image plane the resolution along the lateral axis will be worse than the resolution in the transverse direction. At the relatively low intensities typically employed in multiphoton microscopy, the diffraction-limit at full width at half maximum (FWHM) of an observed point source in the transverse direction can be obtained to good approximation by Eq (1) [28],

$$r_{transverse,MP}=\frac{0.61\lambda_{EX}}{\sqrt{2}NA}$$ (1)

where *r*_*transverse*_ is the resolution, λ_EX_ is the excitation wavelength, and NA is the numerical aperture of the objective lens. Similarly, the lateral (i.e. in the direct of laser propagation) resolution is obtained by the Eq (2)

$$r_{lateral,MP}=\frac{0.626\lambda_{EX}}{n-\sqrt{n^{2}-{\left(NA\right)}^{2}}}$$ (2)

where *n* is the refractive index of the medium. Note that the diffraction limits in both the transverse and lateral directions are generally reduced in multiphoton microscopy compared to a confocal microscope operating at the same excitation wavelength by a factor of $\sqrt{2}$, though in practice, confocal microscopy tends to use much shorter wavelengths than multiphoton microscopy, resulting in superior resolution despite this.

## Method and materials

### Microscope configuration

**Fig 1:**
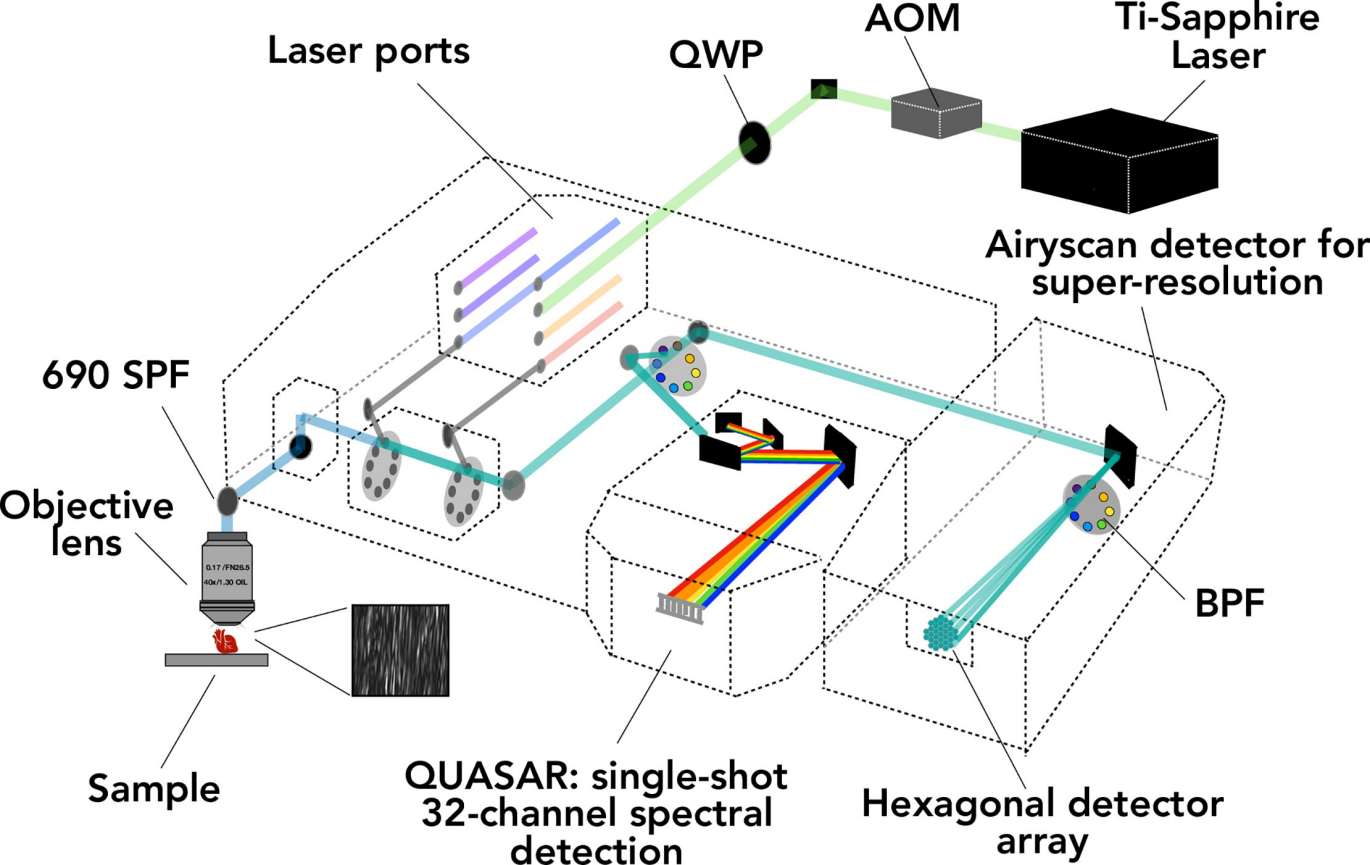
Schematic of the multi-photon Zeiss LSM 880 optical system. Laser pulses are generated by the Ti:Sapphire Chameleon Ultra II laser source at 800 nm with a power of 4W. The pulses are passed through an acoustio-optic modular (AOM) which allows variable intensity of light to pass through. The pulses are then circularly polarized with a quarter wave plate (QWP). The light is directed into the back port of the Zeiss LSM 880 microscope body, where it is focused onto the sample using objective lenses. The backward directed light passes through a 690 short pass filter (690 SPF) then through the confocal pinhole. The introduction of a high reflective mirror into the light path directs the light toward the QUASER detector, where the light is dispersed in a series of gratings, then sent to a movable slit that controls the size and position of the detection window. If the high reflector is not inserted into the light path, then the light will continue through the bandpass (BPF) filter before reaching the Airyscan detector.

Our optical system consisted of a commercially available Zeiss LSM 880 (Carl-Zeiss-Straße 22, 73447 Oberkochen, Germany) inverted confocal microscope, equipped with an Airyscan detector. To an external port on the Zeiss LSM 880 we connected an external Coherent Chameleon Ultra II femtosecond Ti:sapphire laser (Coherent, 5100 Patrick Henry Dr, Santa Clara, CA 95054, USA). Such a laser configuration was approved and installed under the warranty purchased from Zeiss. The optical path for the system is shown in Fig 1. In brief, the Ti:sapphire laser produces tunable pulses at an 80 MHz repetition rate with up to 4W of power in the range from 680–1050 nm. The pulses were passed through an acoustic optical modulator (Carl-Zeiss-Straße 22, 73447 Oberkochen, Germany), followed by a broadband quarter wave plate (AQWP10M-980, Thorlabs) to generate a circularly polarized light. This light was then directed into the back port of the microscope. For our experiments, an excitation wavelength of 800 nm was selected as this is known to produce a strong SHG response at 800 nm in fibrillar collagen [6,8]. Imaging was conducted with a 63x oil immersion lens (Plan-Apochromat SF25, Zeiss) with numerical aperture (NA) of 1.4. The measured transmission of light from the laser source at this wavelength to the sample is 17%. For our experiments, the laser power was typically set to 2.2% of the laser maximum, yielding 15 mW of 800 nm incident light on the sample. Excess laser light was blocked by a 690 shortpass filter. The confocal pinhole was set to its maximum, 600 μm for all measurements. In our inverted system, all light is collected in the backward direction, either to the internal GaAs detectors, or to the Airyscan detector, depending on the insertion of a mirror into the optical path. It is important to note that for SHG, the inverted microscope configuration leads to weaker signal collection compared to what would be available with an upright microscope since SHG is predominantly forward-propagating, though this effect is minimized by using circularly polarized light, which increases the amount of backward directed SHG from collagen.

#### Microscope software settings

The Zeiss LSM 880 has three internal detectors for which the range can be tuned with high precision using an internal diffraction grating system. One channel was used to collect light in the range of 385–415, corresponding to the SHG of the laser; the other was set to the range of 500–600 nm, which corresponds to the peak of the two-photon excitation fluorescence (TPEF) of biomolecules within the tissue, including predominantly elastin [29]. The detector gains were set to 700 for the GaAs detector. For Airyscan images, the gain was set to 1000. Due to the differences in detector design, the gains between the GaAs and Airyscan are not directly comparable, and the gains were chosen to optimize the signal intensity. Images were taken with 4x signal averaging. The Airyscan detector itself consists of a 32-element detector array, with each detector having a size of approximately 0.2 Airy unit. The detector has an internal calibration system to ensure that the light optimally fills the detector array. The Airyscan detector provides increased resolution through two means. First, the resolution is improved by a factor of approximately 1.3 due to the 0.2 Airy unit pinhole size of each of the detectors. Furthermore, the Airyscan gains additional information due to pixel reassignment [23]. In essence, the detector has additional information about the spatial distribution of the light reaching any given detector element, as the detector scans across the sample. As a point source is scanned, its position will fall on different detector elements. By reassigning the point source to the same element in the detector, the SNR can be dramatically increased, and upon deconvolution, the rendered image will have a greater resolution than the pinhole alone.

#### Microscope customization

The Airyscan system is not connected to the spectral grating system, so it is not possible to specify precisely the channel bandwidths of the detectors. Instead, conventional filters must be inserted into the optical path to block the light. For imaging fibrillar collagen, we introduce a 400–20 band-pass (BP) filter (FF01-400/40-10-D-EB, Semrock) into the optical path. This filter is nonstandard with the purchase of the commercial Airyscan system, but can be mounted into a Zeiss filter holder and installed by Zeiss to comply with the microscope warranty. To detect TPEF, we used a 460–505 BP + 525 LP filter, which was included with the Airyscan package, into the beam path to exclude the SHG.

### Image acquisition and processing

SHG images were acquired at an optical zoom of 2.5, corresponding to a field of view of 54x54μm with 2048x2048 pixels, yielding 26 nm per pixel, or at zoom 10, corresponding to a 13x13 μm field of view with 1024x1024x pixels, yielding 13nm per pixel. Z-stacks were collected for both the GaAs and Airyscan detector at zoom 10 with a 512x512 field of view, using a 40 nm step size. Raw Airyscan images were processed using the Zen Black software’s Airyscan Processing function in 2D mode, with a kernel weight of 6. TPEF images were collected in the manner described above at zoom 2.5 using a laser power of 2% with an optical field of 54x54μm with 2048x2048 pixels.

#### Image analysis

Cross-sections of fibers were extracted using the Zeiss Zen Blue analysis software, and fit to a Gaussian distribution using the ImageJ Curve Fitting tool. For the Z-stacks, the Zen Blue rotate stack function was used to create images in the x-z plane, from which profiles could be extracted in the same manner. Fibers chosen for analysis were selected on the basis of being sufficiently isolated so as to be able to achieve a good Gaussian fit, and with a signal to noise of at least 8 to 1. Several fibers were selected from each field for analysis, and three arbitrarily selected fields of view from three different tissue samples were analyzed. We examined the effect of oversampling, under-sampling, and Nyquist sampling on the image quality and uncertainty in the resolution (Sheet C in S1 Dataset). The errors in the fits were calculated using the standard formula $\Delta FWHM=FWHM/(\sqrt{2N-2})$ where N is the number of points used in the fit. Using the oversampled parameters, the error in the full-width-half-maximum could be reduced to 18 nm, an error of approximately 12%, which generally resulted in the best fits; at Nyquist sampling, the error is 22 nm, or 15%. Under-sampling the sample can produce unacceptably large errors: For example, at 44 nm/pixel, the error is 30 nm, over 20%. We identified 25 distinct fibers in a field of view and systematically measured the thickness of each. The thicknesses were tallied in a histogram in bins 10 nm wide centered at 120 nm, 130 nm, etc. The SNR values presented in this work were obtained using the NoiSee plugin for Fiji [30].

#### Directionality analysis

This analysis is based on Liu’s work [31], which served as a base for the development of the directionality plugin for Fiji. Liu introduced a new method for linear pattern extraction and directionality analysis based on Fourier spectrum analysis. For a square image, structures with a preferred orientation generate a periodic pattern at 90° orientation in the Fourier transform of the image compared with the direction of the objects in the input image. Then the image is chopped into square pieces, and their Fourier power spectra are computed. The latter are analyzed in polar coordinates, and the power is measured for each angle with the spatial filters proposed in [31]. Angles are reported as follows: 0° indicates the east direction, and direction moves counterclockwise with increasing angles.

### Sample preparation

De-identified samples of normal human cardiac ventricle muscle and human lung tissue were obtained from the Centre for Heart Lung Innovation’s Cardiovascular biobank and lung registry. All tissues were obtained with informed patient consent under the approval of Providence Health Care Research Ethics board. The heart and lung tissues were fixed in neutral buffered formalin, then infiltrated with low melting point paraffin using a tissue processor (Model ASP6025, Leica, Ontario, CA,). 5μm thick sections were taken using a microtome, followed by dewaxing with xylene and graded alcohols (100%, 90%, 80%, 70%). The sample was then stained with hematoxylin and cover-slipped before imaging. The study was approved by the Providence Health Care Research Ethics Board (H13-02173) at the University of British Columbia.

#### Fibroblast-seeded collagen gels

**C**ollagen I gels were made according to the protocol first established by Bell and colleagues [32] with a slight adaptation as previously described [33]. Type I Rat tail collagen in 0.02N acetic acid (Corning) was diluted to 0.4mg/mL in DMEM (Lonza). 1mL of the diluted collagen I solution was added to 12 well tissue-culture plates previously coated with 1% BSA (Sigma) in DMEM, and allowed to polymerize for 16 h at 37°C. Polymerized collagen 1 gels were carefully detached from the sides of the culture plate to ensure they were free-floating. Primary airway fibroblasts (PAFs) that had been obtained using an outgrowth technique as previously described [34] and grown to confluence were trypsinized at passage 2 and seeded on top of the gels at a density of 40,000 cells per well. Primary airway fibroblasts contracted collagen I gels over 72 hours after which gels were fixed with 4% paraformaldehyde and imaged with the multiphoton microscopy.

## Results

### Super resolution imaging of collagen fibers in human biological samples

Samples with a high content of fibrillar collagen are ideal for the demonstration of SR-SHG imaging since the typical fiber size of fibrillar collagen is 50-200nm, which is below the diffraction limit of conventional SHG. Here, we used formalin-fixed, paraffin embedded samples of unstained human cardiac papillary muscle and lung airway tissue to demonstrate the capacity of the SR-SHG technique on thin-slice (5μm) biobanked specimens. We also analyzed 3-dimensional collagen gels, which are used as an *in vitro* model system to study how tissue stromal cells such as fibroblasts maintain the homeostasis of the ECM environment [10,32].

**Fig 2:**
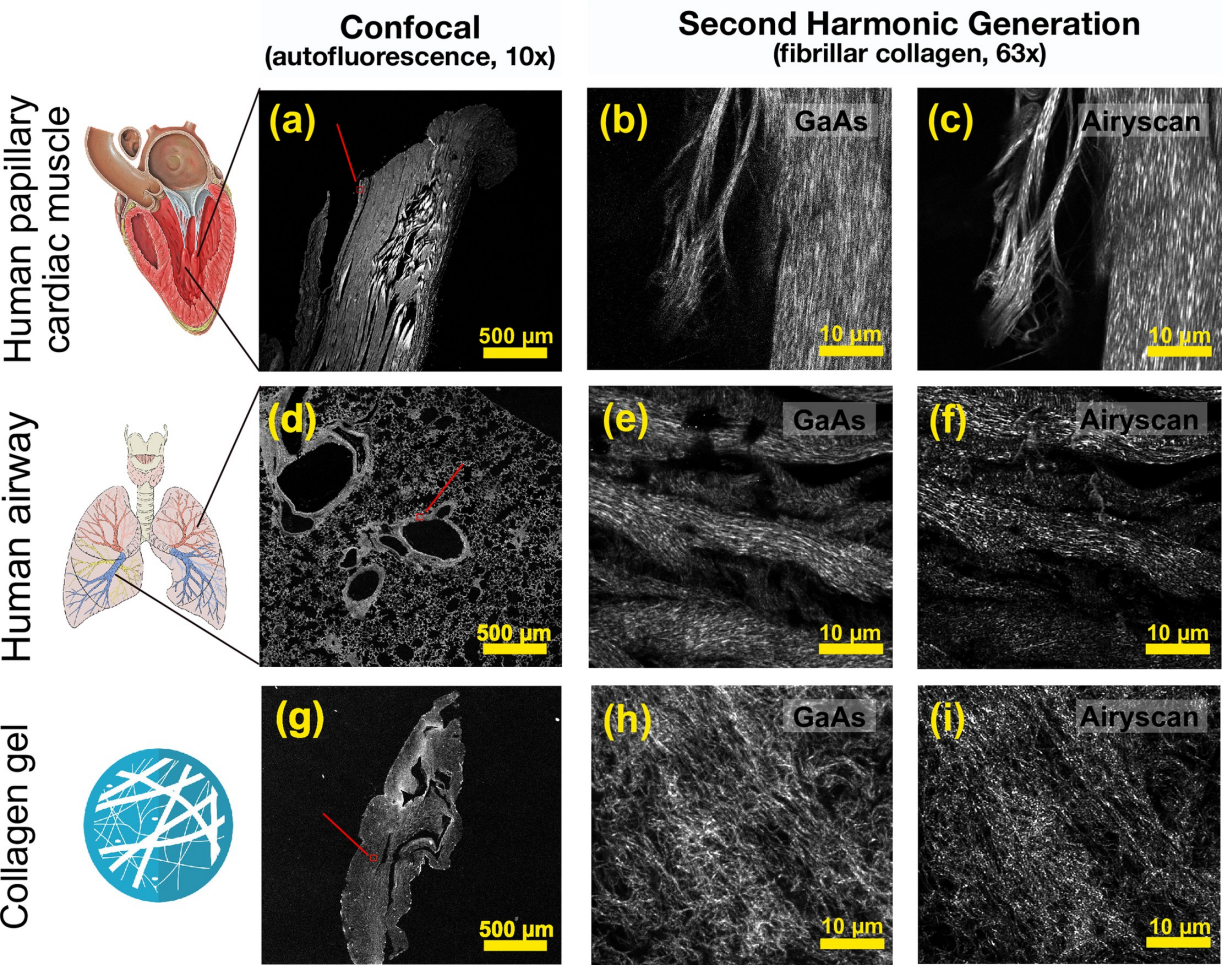
Comparison of SHG images of collagen fibers from biological samples using standard GaAs and Airyscan detectors. Human papillary heart muscle was imaged using (a) autofluorescence from a 488 nm confocal laser source, and SHG using a (b) GaAs detector and (c) Airyscan detector; human airway tissue was imaged using (d) autofluorescence from a 488 nm confocal laser source, and SHG using a (e) GaAs detector and (f) Airyscan detector. Collagen gels were imaged using (g) autofluorescence from a 488 nm confocal laser source, and SHG using a (h) GaAs detector and (i) Airyscan detector. Fields of view: Confocal, 2.4mm x 2.4mm; SHG, 54 μm x54μm. Objective lens: Confocal, 10x; SHG, 63x oil immersion. Acknowledgments to Carl Jaffe, MD, cardiologist (heart image) and Patrick J. Lynch, medical illustrator (heart & lung image).

A wide field image of human papillary heart muscle is shown in Fig 2A, generated by autofluorescence using a 488 nm confocal laser source at 2% power. Images were taken using a 10x lens, and a 3x3 tile scan to generate a 2.4mm x 2.4mm field of view. High magnification SHG imaging of the human papillary heart muscle using the GaAs detector (Fig 2B) and Airyscan (Fig 2C) were scanned in the lateral direction with a laser power of 2.2% (15mW at the sample) at 800nm using a 63x lens with a 54μm x 54μm field of view (2048x2048). The images from airway lung tissue (Fig 2D–2F) were generated using the same protocols. The 3-dimensional collagen gels were scanned in a similar manner using auto-fluorescence (Fig 2G), but a laser power of 8% (54mW at the sample) was used with the GaAs detector (Fig 2H) and Airyscan detector (Fig 2I). Compared to the standard GaAs detector (Fig 2B, 2E and 2H) all images acquired with the Airyscan detector (Fig 2C, 2F and 2I) had significant improvement in resolution and SNR, to enable visualization of collagen fibers.

### Quantitative measurement of collagen fibers using super resolution imaging

**Fig 3:**
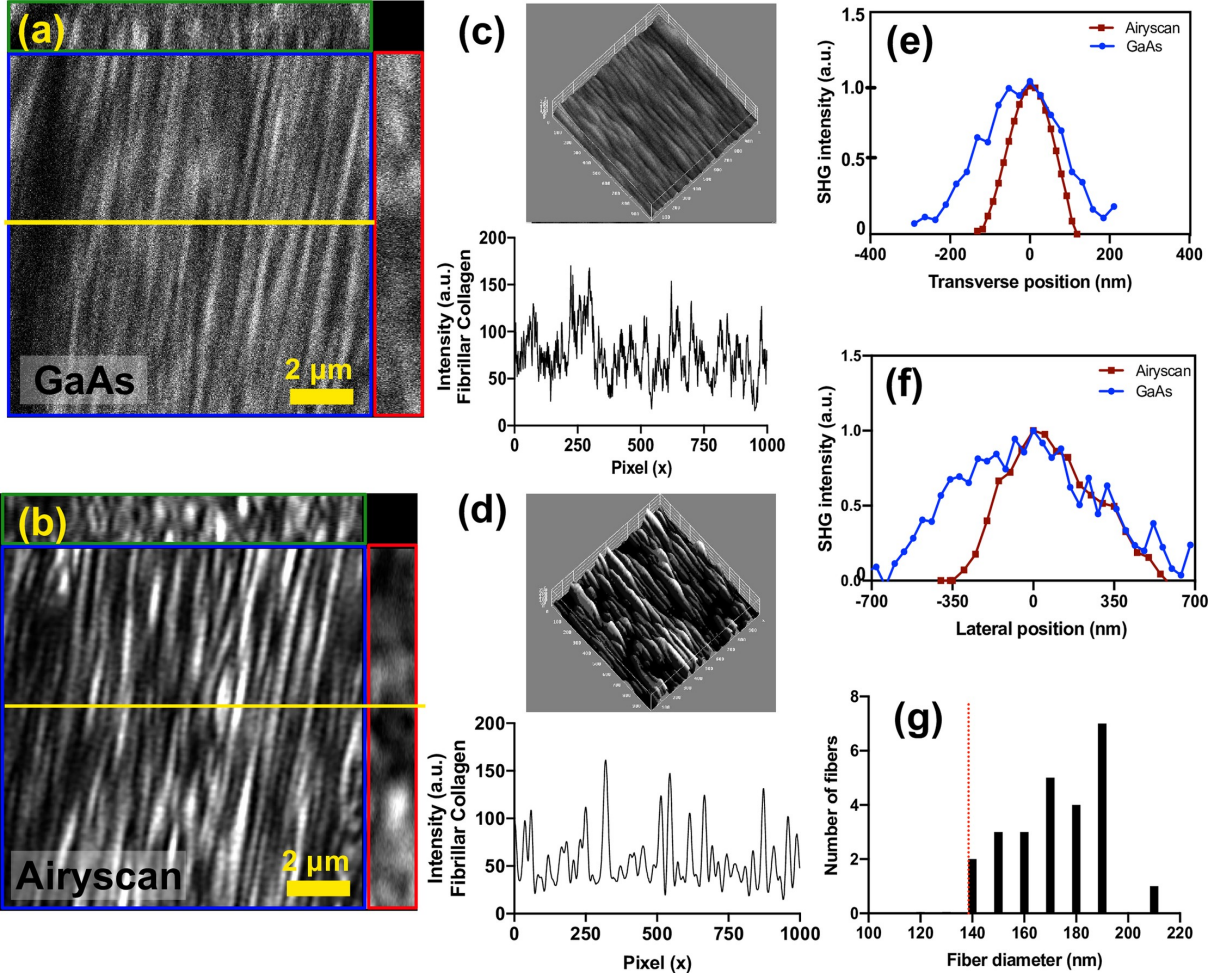
Assessment of collagen fiber dimensions using standard GaAs and Airyscan detectors for SHG imaging. Representative image from 3-dimensional z-stack using (a) GaAs detector or (b) Airyscan detector in 13 μm x 13μm x 2 μm volume, with z-step size of 40 nm. The yellow line across the images represent the location from where the image profile presented in (c) for the GaAs detector and (d) for the Airyscan were obtained from. FWHM thickness measurements of human cardiac tissue using SHG signal in (e) the transverse direction using Airyscan (red) and GaAs (blue) detectors. (f) Representative measurement of fiber thickness of human cardiac tissue in the lateral direction, using Airyscan (red) and GaAs (blue) detectors. (g) Survey of FWHM fiber thicknesses of 25 fibers in human cardiac tissue with Airyscan (black bars) and the theoretical SR diffraction limit is highlighted by the red dashed line at 140 nm.

To determine with precision if the Airyscan detector could surpass the diffraction limit for SHG imaging the papillary heart muscle was scanned in the both transverse and lateral position with smaller field of view (13μm x 13μm), with an oversampled scanning area of 1024 x 1024 pixels, using the standard GaAs detector (Fig 3A) and Airyscan detector (Fig 3B). In the cardiac tissue, the image intensity profile of the fibrillar collagen structures (Fig 3C and 3D) show parallel fibers can be much more clearly distinguished when observing them using the Airyscan detector (Fig 3D), due to mostly improving the SNR of the SHG intensity. In the transverse dimension, the FWHM of the smallest fibers observed measured 260±33nm with the GaAs detector compared to 149±18nm with the Airyscan detector (Fig 3E), yielding an improvement factor of 1.7±0.3x. In the lateral dimension, the FWHM fiber thicknesses measured were 770±100nm with the GaAs detector, and 490±80nm with the Airyscan detector, yielding an improvement factor of 1.6±0.3x (Fig 3F). From Eq (1), the diffraction limit for MP-SHG under these conditions is expected to be 246nm, thus we can conclude that the GaAs detector (260±33nm) in the transverse dimension is diffraction limited, whereas the Airyscan detector (149±18nm) achieves super resolution in the transverse dimension. From Eq (2), the diffraction limit in the lateral dimension is expected to be 520 nm (for refractive index (*n)* equals to 1.5 and numerical aperture (NA) of 1.4, as per Eqs 1 and 2), and hence the GaAs detector is not diffraction limited, and the Airyscan achieves a resolution slightly under the diffraction limit. Using the Airyscan, we measure a range of fiber thicknesses in the transverse dimension between 140–200 nm (Fig 3G), suggesting that ~140nm represents the minimum observable fiber size.

**Fig 4:**
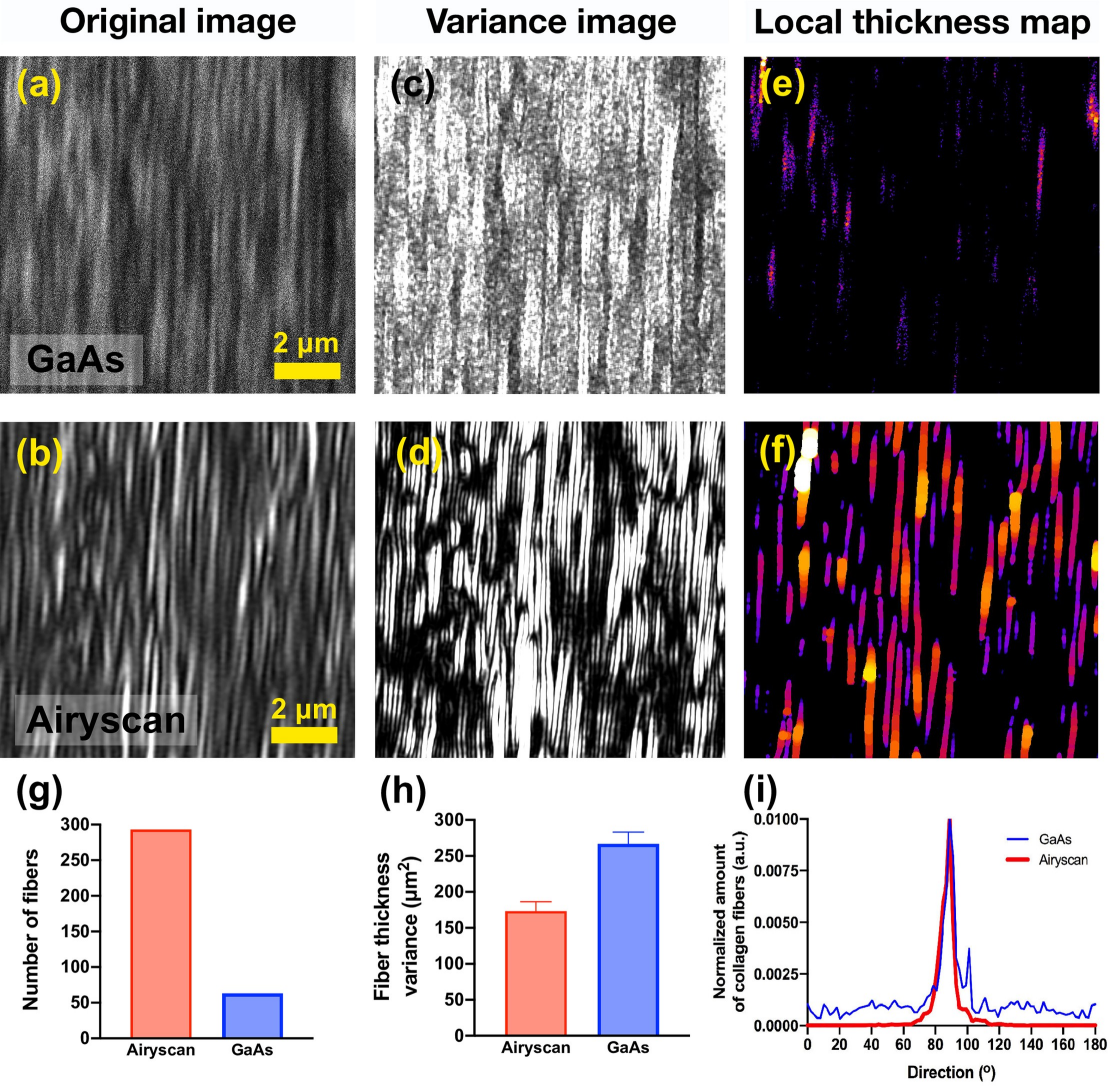
Fiber directionality and variance analysis using GaAs detection and Airyscan detectors. Illustrative SHG images of collagen fibers from human papillary cardiac muscle using (a) GaAs detector or (b) Airyscan detector in 54 μm x54 μm field of view. Illustrative examples showing collagen fibers boundaries obtained from the SHG intensity signal variance using (c) GaAs detector and (d) Airyscan detectors. Colour-coded thickness distribution map of the collagen fibers imaged using (e) GaAs detector or (f) Airyscan. From these maps it is possible to obtain the (g) number of fibers successfully identified; (h) fiber thickness variance, and (i) the amount of fibrillar collagen fibers detected at specific directions, using GaAs detector (blue boxes and line) or Airyscan (red boxes and line).

In addition to accurately measuring fiber thickness, in Fig 4 we clearly demonstrate that compared to the GaAs detectors (Fig 4A) the images obtained using the Airyscan detector (Fig 4B) enables identification of collagen fiber structures with a higher SNR. The SHG signal intensity using the GaAs detector was 81.6±49.6 respectively, yielding a SNR of 1.64. Using the Airyscan detector the mean SHG signal intensity was 75.7±24.9, yielding an SNR of 3.03. To visualize such differences, we compared the pixel intensity variance of the detected SHG signal using both detectors. The image variance was obtained by replacing each pixel with the neighborhood variance, which highlights the edges of the fiber structure. The image variance clearly shows how the increased SNR obtained using the Airyscan detector (Fig 4D) makes it possible to visualize the exact boundaries of many fibers within the image compared to the image obtained using the GaAs detector (Fig 4C).

The SNR is important as it enables automated image processing methods to quantify fiber structures. To demonstrate this, we calculated a local thickness map of the GaAs (Fig 4E) and Airyscan (Fig 4F) images, which enables one to derive features such as fiber number, thickness and directionality. As shown in Fig 4G, the number of collagen fibers detected was greater in the Airyscan image compared to the GaAs detector. The variance of collagen fiber thickness was lower using the Airyscan detector compared to the GaAs detector (Fig 4H). Lastly, the variance in fiber directionality was lower in collagen fibers imaged using Airyscan detector versus the GaAs detector (Fig 4I).

### Super resolution imaging of elastic fibers in human biological samples

**Fig 5:**
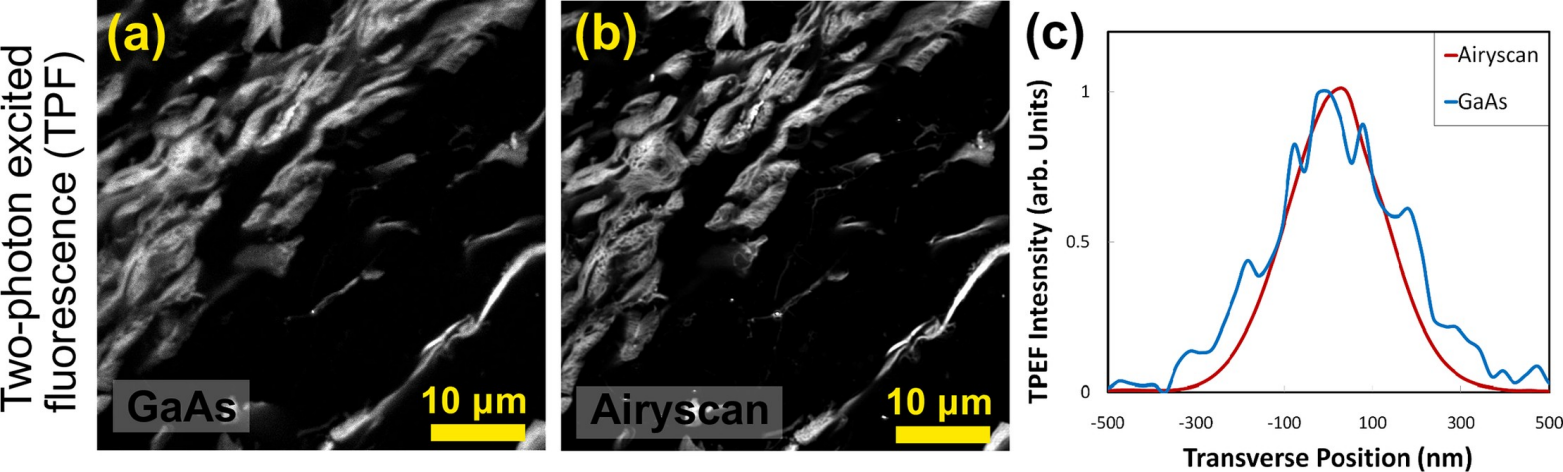
Comparison of TPEF images of papillary heart muscle using GaAs detection and Airyscan detectors. TPEF images using (a) GaAs detector or (b) Airyscan detector. (c) TPEF signal in the transverse direction using Airyscan (red) and GaAs (blue) detectors. Field of view: 54 μm x54μm. Objective lens: 63 oil immersion.

Using the same laser power (2.2%), 63x lens, and optical field of 33x33μm (1024x1024 pixels) the TPEF signal was collected from the papillary heart muscle and again compared to the GaAs detector (Fig 5). Representative images of TPEF imaging of the papillary tissue, collected concurrently with the SHG at the same settings, were collected using the GaAs and Airyscan detection systems (Fig 5A and 5B). We were not able to detect individual elastin fibers in the TPEF. Nonetheless, the Airyscan also produced a notable improvement in the quality of TPEF signal, with the smallest observable structures measured with a lateral diameter of 330±40nm with the GaAs detector and 230±30nm with the Airyscan detector, yielding an improvement factor of 1.4±0.3x (Fig 5C).

## Discussion

Our results demonstrate the viability of the commercial Airyscan for SR-MP microscopy, without the need of custom-built or complex customized systems. Specifically, in the transverse dimension, Airyscan provides a substantial improvement in image quality and SNR compared to traditional GaAs detectors, resulting in a 1.7±0.3x improvement in resolution beyond the diffraction limit for SHG. We conclude that the performance of Airyscan is not adversely affected for MP as compared to confocal microscopy, as the expected improvement in resolution for confocal microscopy is also 1.7x [19,23]. The improvement of SNR is likely due to the design of the Airyscan detector, which uses a detector array of high-quality detectors with an effective size of 1.25 Airy unit confocal pinhole, but because each detector is only 0.2 Airy units in size, the detector is able to reject out of focus light far more effectively that a 1.25 Airy unit pinhole. The SNR results we report for multiphoton microscopy are consistent with SNR improvements reported with the Airyscan in confocal applications [23, 24]. The system performed somewhat less effectively for SR measurements in the lateral dimension, only yielding an improvement of 1.6±0.3x in resolution. As discussed previously, in the lateral dimension, the excitation volume is much more sensitive to the intensity of the laser than in the transverse dimension, and it is likely that we were unable to achieve a diffraction-limited result as a consequence, but we could not explore this possibility as increasing the laser intensity significantly would exceed the damage threshold of our specimens. It is also possible that the light experiences greater scattering in the lateral dimension at increasing depth, as suggested by the asymmetric lineshape in Fig 3F, which results in a loss of resolution. The diffraction limited measurements generally are dependent on the experimental conditions, including alignment, stability of the optical system, the refractive index of the sample, and it may not always be possible to achieve the diffraction limit in all specimens due to these factors.

In the collagen gels, we were able to achieve a diffraction limited measurement using the conventional detector, but only saw an improvement of 1.2±0.3x in the Airyscan system. The difference in these measurements likely has to do with the typical fiber size in the respective samples. We note in Fig 3G that approximately 10% of observed fibers in the cardiac tissue were diffraction limited, whereas the sizes of the remaining fibers were measured accurately in a distribution of sizes between 150–200 nm. Conversely in the gels, the smallest observed fibers were measured at 213 nm (Sheet A in S1 Dataset), and with a range of thicknesses up to 320–360 nm (Sheet B in S1 Dataset).We note that the thickness of collagen fibers produced in such gels is sensitive to the protocol used to generate them [35], so it is likely that we are simply observing fibers of larger thickness in the gels as compared to the tissues.

We did not achieve a diffraction limited measurement in the TPEF for the GaAs detector, and only observed an improvement of 1.4±0.3x using the Airyscan detector. The TPEF producing structures in our specimens did not have the same dense fiber structure as the SHG collagen fibers, so a systematic study of individual fiber thicknesses was not possible, and thus it could not be ascertained if diffraction limited measurements can be achieved with this technique.

The morphology and distribution of ECM fibers are affected in several diseases, such as cancer, atherosclerosis, asthma, skin disorders [36–39], and the ability to accurately quantify and identify these changes bring unique perspectives related to disease mechanisms. In fibrosis, the ECM fibers are disorganized and present significant changes in thickness and orientation [40–42], and access to such information has been bringing a new perspective in understanding for example, lung and cardiovascular diseases, such as asthma [10], and atherosclerosis [9].

The direction which the fibers are oriented varies drastically depending on the sample and its orientation, as well as the application and question to be answered [43]. When comparing the potential of detecting fiber structures, illustrated by the images of human papillary heart muscle (Fig 4A and 4B) and their orientations (Fig 4K), once again it is clear the advantage of SR-SHG images. The “amount” of identified collagen fibers is related to the percentage of fibers successfully identified within an image which are oriented along a certain preferred direction. This value is significantly affected when comparing the data obtained using the GaAs detector with the Airyscan, from 15% to 78% of the fibers, respectively. A higher amount of fibers detected by the Airyscan provides more accurate information related to their preferred direction of ECM fibers, which often is an important endpoint in biological studies. Fiber thickness variance obtained from SR-SHG images (Fig 4J) show the Airyscan provides more accurate measurements of the fiber dimension when compared to the GaAs detector, having a lower variance thickness value. Finally, we demonstrate that the Airyscan detector provides more accurate information which serves as base to other image processing strategies, such as object identification. We show that the number of objects–or, in this case fibers, identified by the Airyscan detector is significantly higher than what has been obtained by the GaAs detector (318 and 63, respectively).

When comparing the quantitative results of images using the Airyscan compared to the GaAs detectors, one can notice the higher SNR and lower variance of the several parameters calculatedImages with high signal-to-noise ratio allow the accurate identification of fibrillar structures within the field of view, where simple threshold methods are suitable to proper identify these structures with reduced error. Therefore, parameters such as directionality and thickness become more precise and more powerful to be adopted in several biological applications, where distinguishing the characteristics of collagen remodelling are important features when aiming to distinguish between diseases grades or severity levels.

In conclusion, the Airyscan system provides a quantitative improvement to the resolution of multiphoton imaging beyond the diffraction limit. Importantly, unlike other MP-SR alternatives [7,8,15,18] multiphoton imaging can be incorporated seamlessly into a commercial confocal Airyscan system without substantial incremental cost, complex optical components, loss of warranty or the requirement of extensive sample preparation techniques. Airyscan thus provides an ideal candidate to biomedical imaging laboratories that require SR imaging capabilities to image biological molecules such as collagen and elastin that are smaller than the diffraction limit for multiphoton microscopy.

## Supporting information

S1 Dataset Supplemental dataset. Shows additional and raw data. Sheet A: Representative measured thickness of a collagen gel fibre using GaAs and Airyscan. Sheet B: Distributions of measured thicknesses of collagen gel fibres using Airyscan. Sheet C: Examining the effects of Undersampling and Oversampling in SR measurements. Sheet D: Measured thickness of human airway tissue in SR vs GaAs. Figs 3E, 3F, 3G and 5C: Underlying raw data accompanying the indicated figures in the main text.
(XLS)
